# Interpretable artificial intelligence for classification of alveolar bone defect in patients with cleft lip and palate

**DOI:** 10.1038/s41598-023-43125-7

**Published:** 2023-09-22

**Authors:** Felicia Miranda, Vishakha Choudhari, Selene Barone, Luc Anchling, Nathan Hutin, Marcela Gurgel, Najla Al Turkestani, Marilia Yatabe, Jonas Bianchi, Aron Aliaga-Del Castillo, Paulo Zupelari-Gonçalves, Sean Edwards, Daniela Garib, Lucia Cevidanes, Juan Prieto

**Affiliations:** 1 Department of Orthodontics and Pediatric Dentistry, University of Michigan School of Dentistry, Ann Arbor, MI USA; 2 Department of Orthodontics, Bauru Dental School, University of São Paulo, Bauru, SP Brazil; 3 Department of Health Science, School of Dentistry, Magna Graecia University of Catanzaro, Catanzaro, Italy; 4 CPE Lyon, Lyon, France; 5 Department of Restorative and Aesthetic Dentistry, Faculty of Dentistry, King Abdulaziz University, Jeddah, Saudi Arabia; 6 Department of Orthodontics, University of the Pacific, Arthur A. Dugoni School of Dentistry, San Francisco, CA USA; 7 Department of Oral and Maxillofacial Surgery, University of Michigan School of Dentistry, Ann Arbor, MI USA; 8 Department of Orthodontics, Hospital for Rehabilitation of Craniofacial Anomalies, University of São Paulo, Bauru, SP Brazil; 9 Department of Psychiatry, University of North Carolina, Chapel Hill, NC USA

**Keywords:** Image processing, Machine learning, Craniofacial orthodontics

## Abstract

Cleft lip and/or palate (CLP) is the most common congenital craniofacial anomaly and requires bone grafting of the alveolar cleft. This study aimed to develop a novel classification algorithm to assess the severity of alveolar bone defects in patients with CLP using three-dimensional (3D) surface models and to demonstrate through an interpretable artificial intelligence (AI)-based algorithm the decisions provided by the classifier. Cone-beam computed tomography scans of 194 patients with CLP were used to train and test the performance of an automatic classification of the severity of alveolar bone defect. The shape, height, and width of the alveolar bone defect were assessed in automatically segmented maxillary 3D surface models to determine the ground truth classification index of its severity. The novel classifier algorithm renders the 3D surface models from different viewpoints and captures 2D image snapshots fed into a 2D Convolutional Neural Network. An interpretable AI algorithm was developed that uses features from each view and aggregated via Attention Layers to explain the classification. The precision, recall and F-1 score were 0.823, 0.816, and 0.817, respectively, with agreement ranging from 97.4 to 100% on the severity index within 1 group difference. The new classifier and interpretable AI algorithm presented satisfactory accuracy to classify the severity of alveolar bone defect morphology using 3D surface models of patients with CLP and graphically displaying the features that were considered during the deep learning model's classification decision.

## Introduction

Cleft lip and/or palate (CLP) is considered the most common congenital craniofacial anomaly by the World Health Organization^1^. Due to the complexity of this craniofacial anomaly, the rehabilitation process of patients with orofacial clefts is based on a multidisciplinary team widely accepted as a standard approach. The challenges include the surgical closure of the cleft, speech and hearing pathology, anteroposterior and transverse deficiency of the maxilla and dentoalveolar irregularities. The bone grafting of the alveolar cleft is an essential part of the treatment protocol of patients with CLP once it promotes the alveolar bone continuity in the cleft side. The secondary alveolar bone graft (SABG) is considered the standard option to restore the alveolar bone once it presents satisfactory clinical outcomes due to the timing of the surgical intervention^2–4^.

The success of secondary alveolar bone graft can be influenced by several factors such as dental development, age, cleft size and timing of orthodontic treatment^5–9^. A systematic review showed that timing of the SABG (prior to the eruption of maxillary permanent canines), surgical material, and presurgical orthodontics are factors related to the success of SABG^6^. However, there was no sufficient evidence to determine if cleft width or volume influences the clinical outcomes of SABG^6^.

Computed tomography and cone-beam computed tomography (CBCT) scans allow a three-dimensional (3D) assessment of alveolar bone defects of patients with CLP^10–13^. The 3D analysis of the alveolar bone defect allows better surgical planning for the graft and can minimize surgical complications^10,14^. However, 3D image analysis can be a complex and time-consuming task. The use of artificial intelligence (AI) in 3D image analysis tools has increased significantly in the last years with the purpose to simplify and increase the efficiency of this process^15–17^. For this reason, AI-based methods for 3D assessments have become more popular and recent developments are being described. A previous study has demonstrated an efficient novel method for automatic estimation of the alveolar bone defect volume of patients with CLP using a convolutional neural network (CNN)^16^. An open-source algorithm for automated segmentation of multiple anatomic skeletal, dental, and soft tissue structures in the craniofacial complex of CBCT scans based on UNEt Transformers of the Medical Open Network for Artificial Intelligence (MONAI) framework was also described with applications for patients with CLP^17^.

With advancements in AI-driven 3D image analysis, clinical decision support systems (CDSS) are being developed to provide support for clinicians during decisions regarding prevention, diagnosis, and treatment planning^18^. Before these tools can be translated effectively to healthcare, there is still a need to understand the exact features that AI is considering during the decision-making process^19^. An interpretable AI algorithm may provide explainable models to demonstrate the features used by the AI during the prediction task, with the potential benefits of enhancing trust and understanding of AI outputs^19^. For this reason, this study aimed to evaluate the performance of an automated 3D classification index for the severity of alveolar bone defects in patients with CLP based on an interpretable AI algorithm.

## Material and methods

This study was approved by the Institutional Review Board of the University of Michigan School of Dentistry (HUM00222338) and all methods were performed in accordance with the relevant guidelines and regulations. Informed consent was waived by the Institutional Review Board of the University of Michigan School of Dentistry. The sample consisted of secondary data analysis of 194 de-identified cone-beam computed tomography (CBCT) scans of patients with cleft lip and palate acquired in three different university centers (University of Michigan—School of Dentistry, Hospital for Rehabilitation of Craniofacial Anomalies—University of São Paulo and University of Pacific—Arthur A. Dugoni School of Dentistry). The eligibility criteria included: patients with unilateral or bilateral cleft lip and palate or cleft lip and alveolus, mixed or early permanent dentition, and CBCT scan taken for clinical purposes. The exclusion criteria were patients with CBCT scans with artifacts produced by orthodontic appliances.

The 3D analysis was performed using two open-source software packages ITK-SNAP, version 3.8 (https://www.itksnap.org)^20^, and 3D Slicer, version 5.1.0 (http://www.slicer.org)^21^. First, all scans were oriented using the Frankfort horizontal plane perpendicular to the midsagittal plane^22^. Secondly, automatic segmentations of the maxilla were obtained using the automatic multi-anatomical skull structure segmentation (AMASSS) algorithm on Slicer Automated Dental Tools^17^. 3D volumetric label maps (segmentations) and 3D surface models (vtk files) were obtained for all patients.

**Figure 1:**
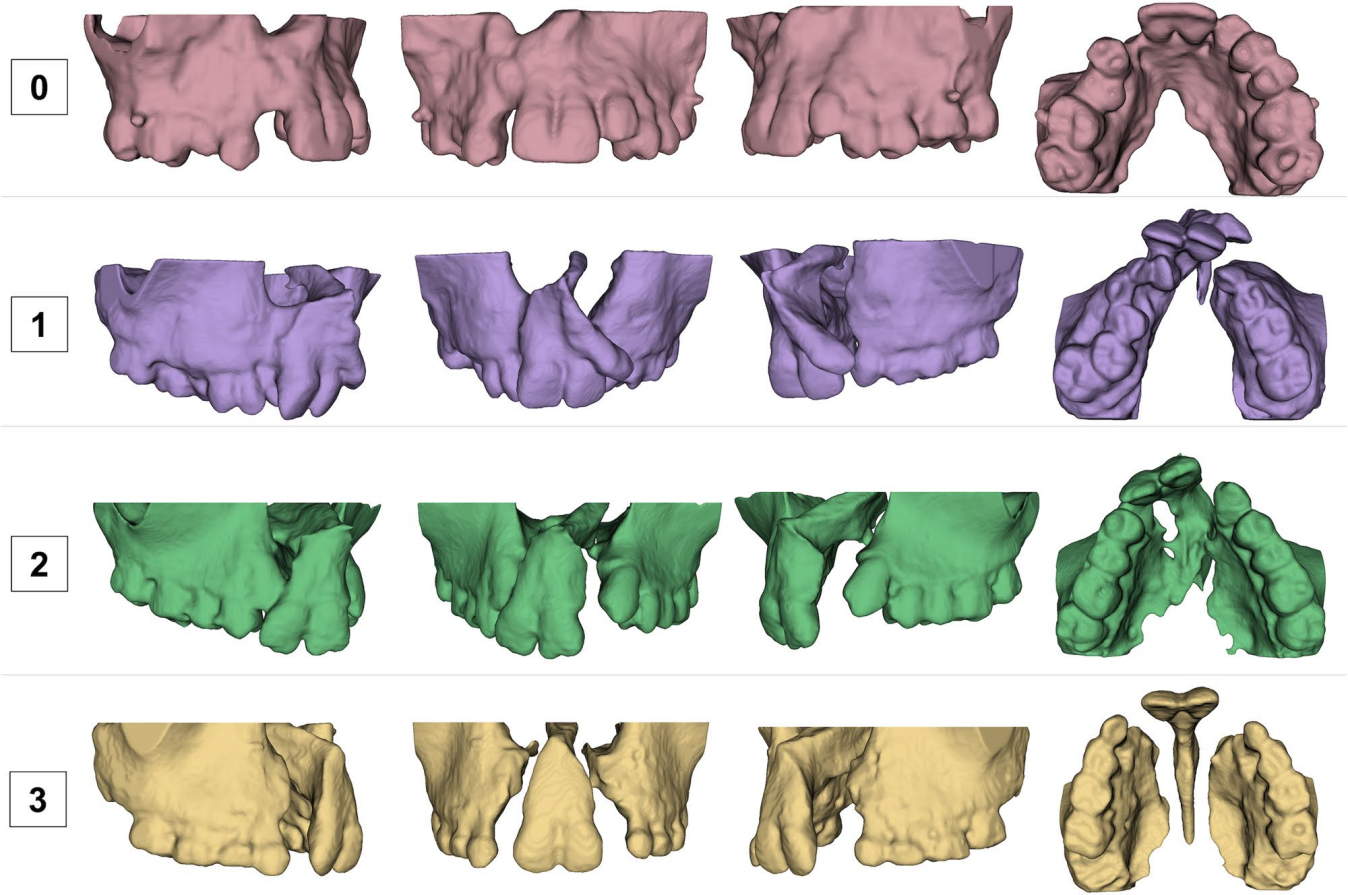
3D surface models of patients classified using the severity index. 0 was considered a lower level of severity and 3 was a greater level of severity.

The shape, height and width of the alveolar bone defect were assessed to determine the severity of the alveolar bone defect and to develop a classification of the severity of the alveolar bone defect using the 3D surface models. The severity of the alveolar bone defect was classified from 0 to 3, where 0 was considered a lower level of severity and 3 a greater level of severity (Fig. 1, Table 1). To determine the ground truth classification index, the sample was classified independently by two calibrated examiners. Examiner 1 repeated all the assessments after a 30-day interval. In cases of no agreement between the examiners’ scores, a third examiner expert in 3D imaging analysis and Orthodontics gave a consensus score. To test the inter and intra-examiner agreement, the Kappa coefficient was used. Inter and intra-examiner reproducibility showed high agreement with kappa values of 0.93 and 0.94, respectively^23^.

**Table 1:** Definition of the severity index for the alveolar bone defect in cleft lip and palate patients.

Index	Description
0	Bone depression in the buccal or at least one wall of bone support in the palatal aspect
1	Complete cleft of the alveolar bone defect in at least one side
2	Projected premaxilla with an alveolar bone defect and some palatal bone continuity or bilateral well aligned defects
3	Projected premaxilla with large alveolar bone defect with a small or no palatal bone continuity

A shape analysis technique was developed and applied in a severity classification task for the alveolar bone defect. The key step in the approach was to extract features that can represent and characterize the 3D shapes in a compact way. This approach is a learning-based method and falls into the multi-view category^24^.

The approach starts using our novel Fly-by-CNN algorithm^25^ by rendering the 3D object from different viewpoints and capturing 2D image snapshots that are then fed to a 2D Convolutional Neural Network (CNN). We aggregate the features from each view via an Attention Layer that encourages the model to select specific features^26^.

In addition to the novel shape classification technique, an explainability approach for 3D shape models called Surface Gradient-weighted Class Activation Mapping (SurfGradCAM), which is an extension of Gradient-weighted Class Activation Mapping (GradCAM)^27^ was implemented. It generates a heatmap that highlights the most important regions of the 3D model. This is achieved by backpropagating the gradients of the output class with respect to the feature maps of the final convolutional layer of each view. The heatmaps are then pooled using a max function and mapped onto the surface of the 3D object.

### Statistical analysis

The data was split as follows: 70% for training, 10% for validation and 20% for testing (5-folds of 38 patients = 190 testing datasets). A fivefold cross-validation was performed. The performance of the algorithm was assessed using the precision, recall, F1 score, and accuracy. A confusion matrix was also performed to allow the visualization of the performance of the algorithm. The agreement between the ground truth classification and the algorithm-predicted classification was represented by the main diagonal of the table. Cells adjacent to the main diagonal (1 diagonal to the right and 1 diagonal to the left) indicate the classification of the severity index was within 1 group difference. A receiver operating characteristic (ROC) curve was also performed to show the sensitivity of the classification model performance.

## Results

**Figure 2:**
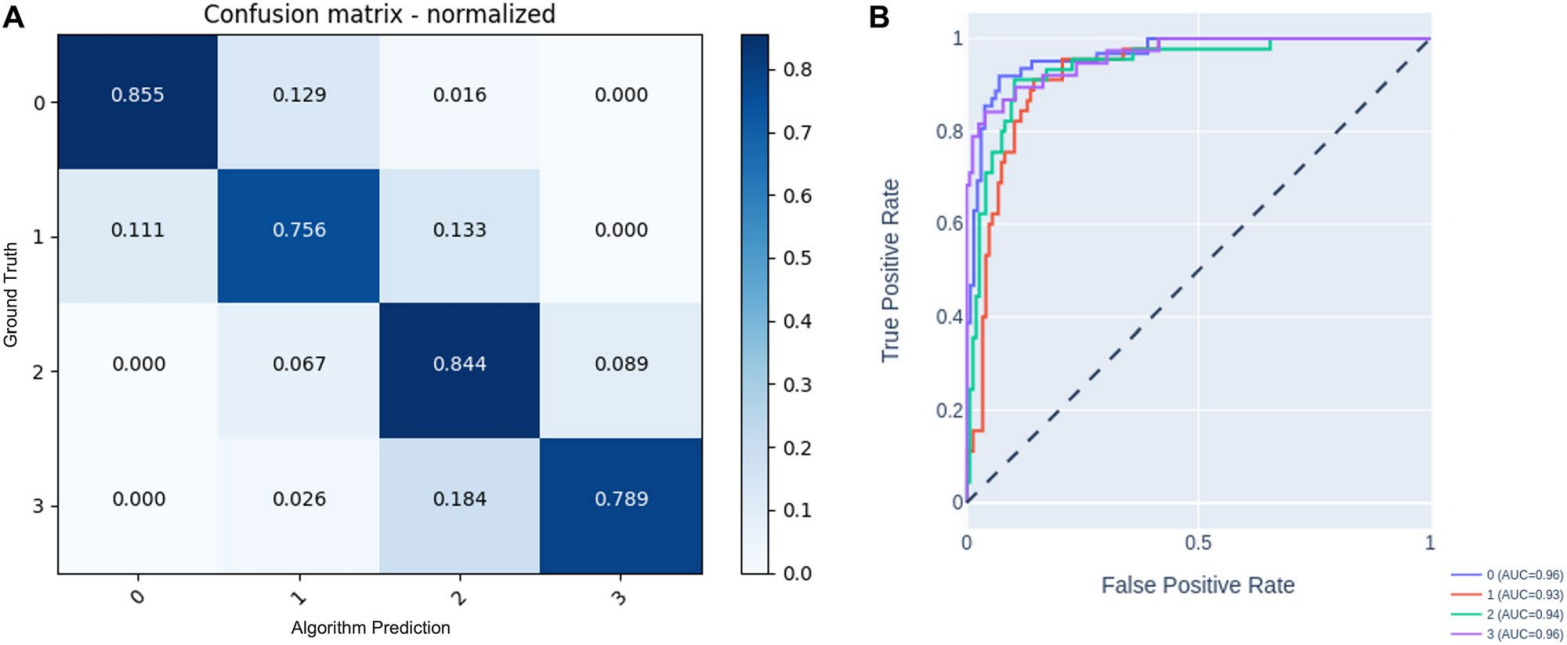
** (A)** Confusion matrix demonstrating the sensitivity of the performance of the classifier algorithm. The rows represent the ground truth as assessed by the consensus between the clinical experts, and the columns represent the algorithm prediction of the severity index. The main diagonal cells (in dark blue) show when the group was classified correctly by the algorithm when compared to the ground truth. The cells in 1 diagonal to the right and 1 diagonal to the left show the differences in the predicted label from the ground truth by only 1 group. (**B**) ROC curve showing the performance of the classification model. High sensitivity in the AI-predictions can be observed for all classes.

The performance of the classifier algorithm is shown in Table 2. The classifier task achieved an overall accuracy of 0.816. In addition, a high overall precision (0.823, SD 0.95), recall (0.816, SD 0.47), F-1 score (0.817, SD 0.59) and AUC (0.948, SD 0.15) were observed for the classifier task (Table 2). The class 0 achieved the highest precision, recall, f-1 score and AUC, while the class 1 achieved the lowest recall, F-1 score and AUC. The confusion matrix shows that the classifier could predict the correct class with an agreement above 97.3% within 1 group difference for the testing data sets (Fig. 2A). The main diagonal cells show when the group was classified correctly by the algorithm when compared to the ground truth (Fig. 2A). A range from 0.75 to 0.85 was found in the main diagonal when comparing the algorithm prediction with the ground truth. High sensitivity can be observed for the AI-predictions in all classes (Fig. 2B).

**Table 2:** Precision and accuracy for the trained model.

Class (n)	Precision (SD)	Recall (SD)	F1-score (SD)	Accuracy	AUC (SD)
0 (62)	0.914	0.855	0.883	n/a	0.96
1 (45)	0.739	0.756	0.747	n/a	0.93
2 (45)	0.731	0.844	0.784	n/a	0.94
3 (38)	0.882	0.789	0.833	n/a	0.96
Total (190)	0.823 (0.95)	0.816 (0.47)	0.817 (0.59)	0.816	0.948 (0.15)

**Figure 3:**
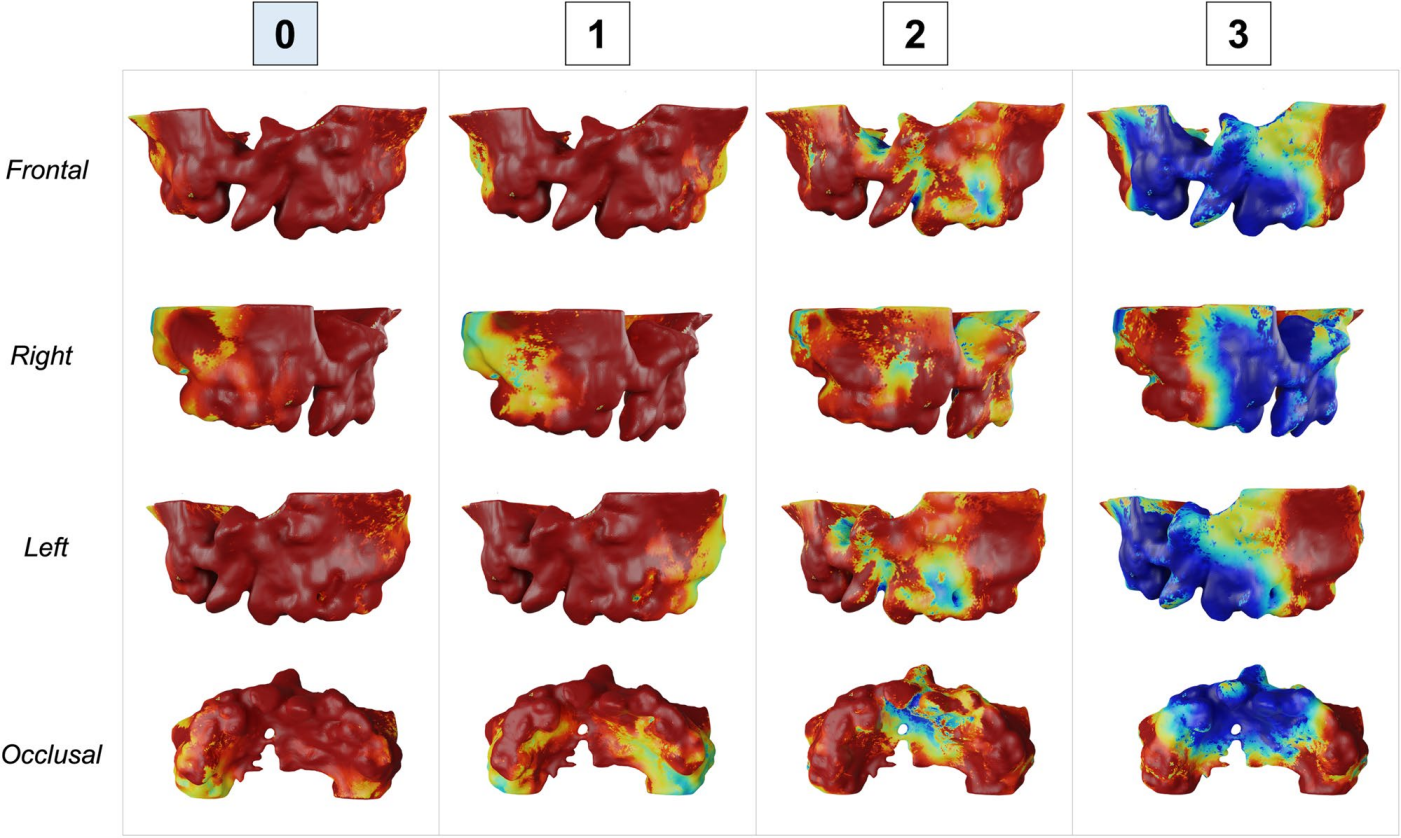
Explainability maps for a 3D surface model properly predicted as a class 0 of the severity index. The heatmaps were generated by each different class (columns) in different view perspectives (rows). In dark red are the most important features considered by the algorithm to output the classification and in blue the less important features. Note that it is necessary to assess the heatmaps of all classes to determine which class highlights in red (or dark red) the cleft features. It can be observed that the algorithm is capable to distinguish the important features in the different classes.

**Figure 4:**
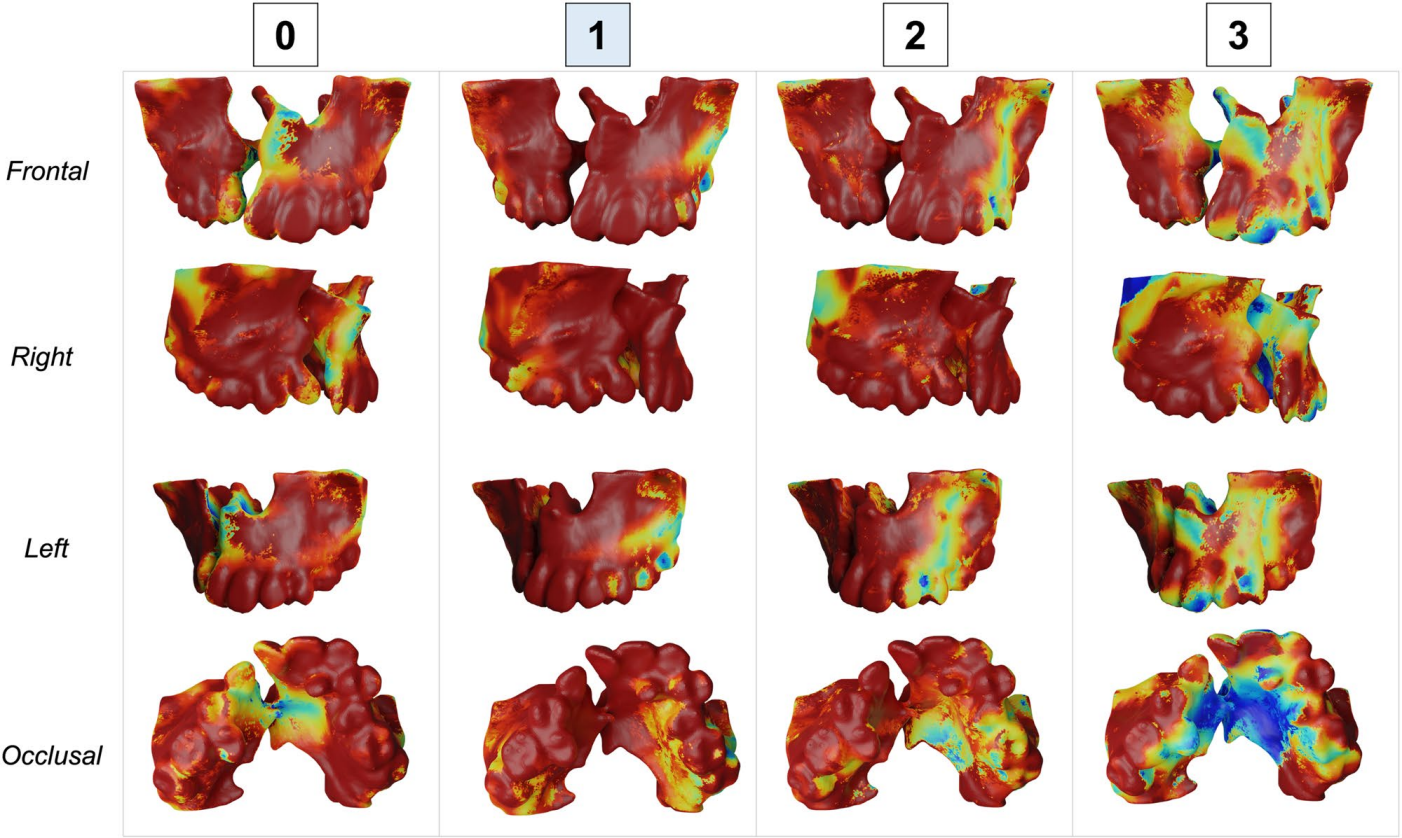
Explainability maps for a 3D surface model properly predicted as a class 1 of the severity index. In dark red are the most important features considered by the algorithm to output the classification, and in blue are the less important features. Note that the heatmap for class 1 focused correctly on the unilateral bone defect.

**Figure 5:**
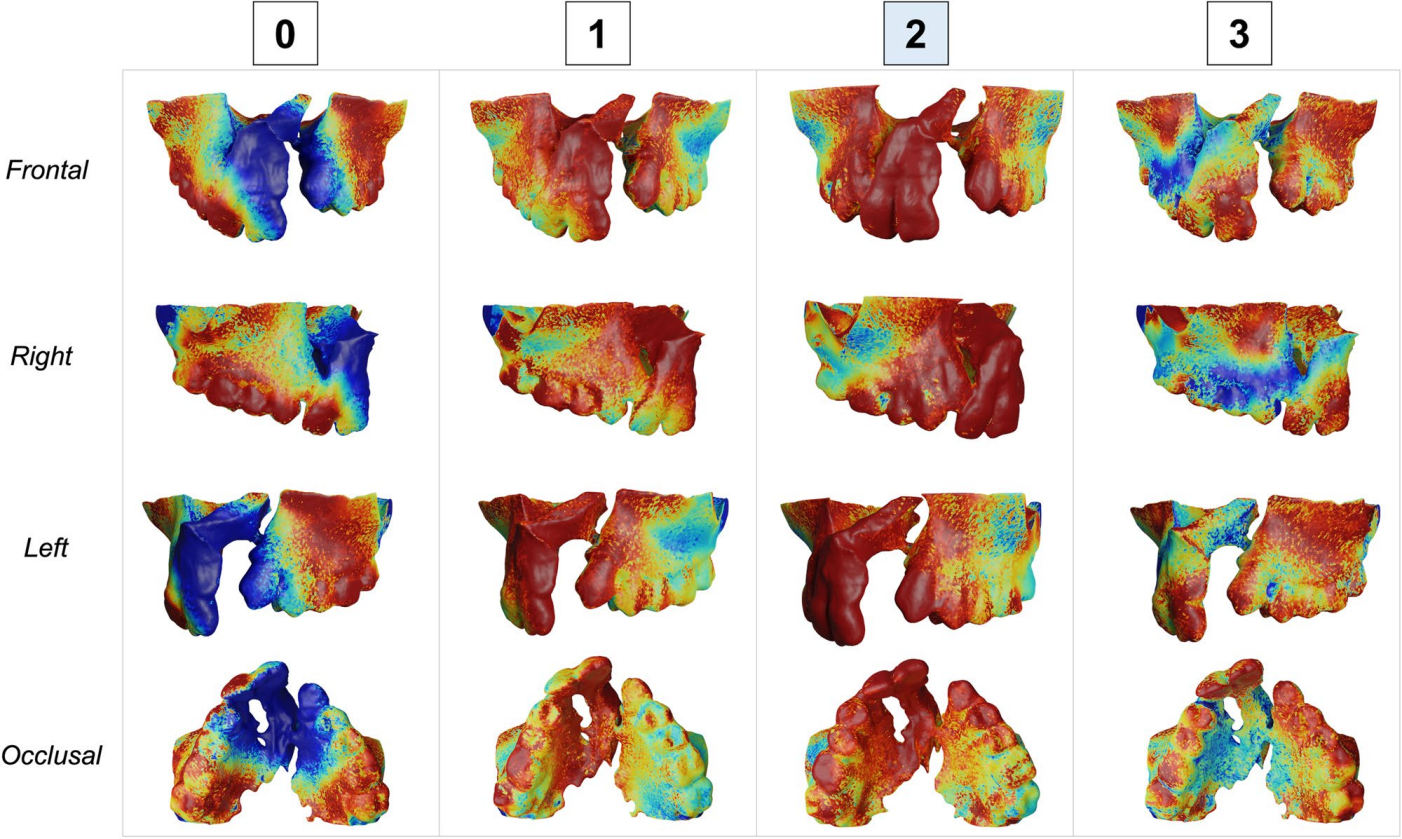
Explainability maps for a 3D surface model properly predicted as a class 2 of the severity index. In dark red are the most important features considered by the algorithm to output the classification and in blue the less important features. Note that the heatmap for class 2 demonstrated the projected pre-maxilla and palatal continuity as important features for this classification.

**Figure 6:**
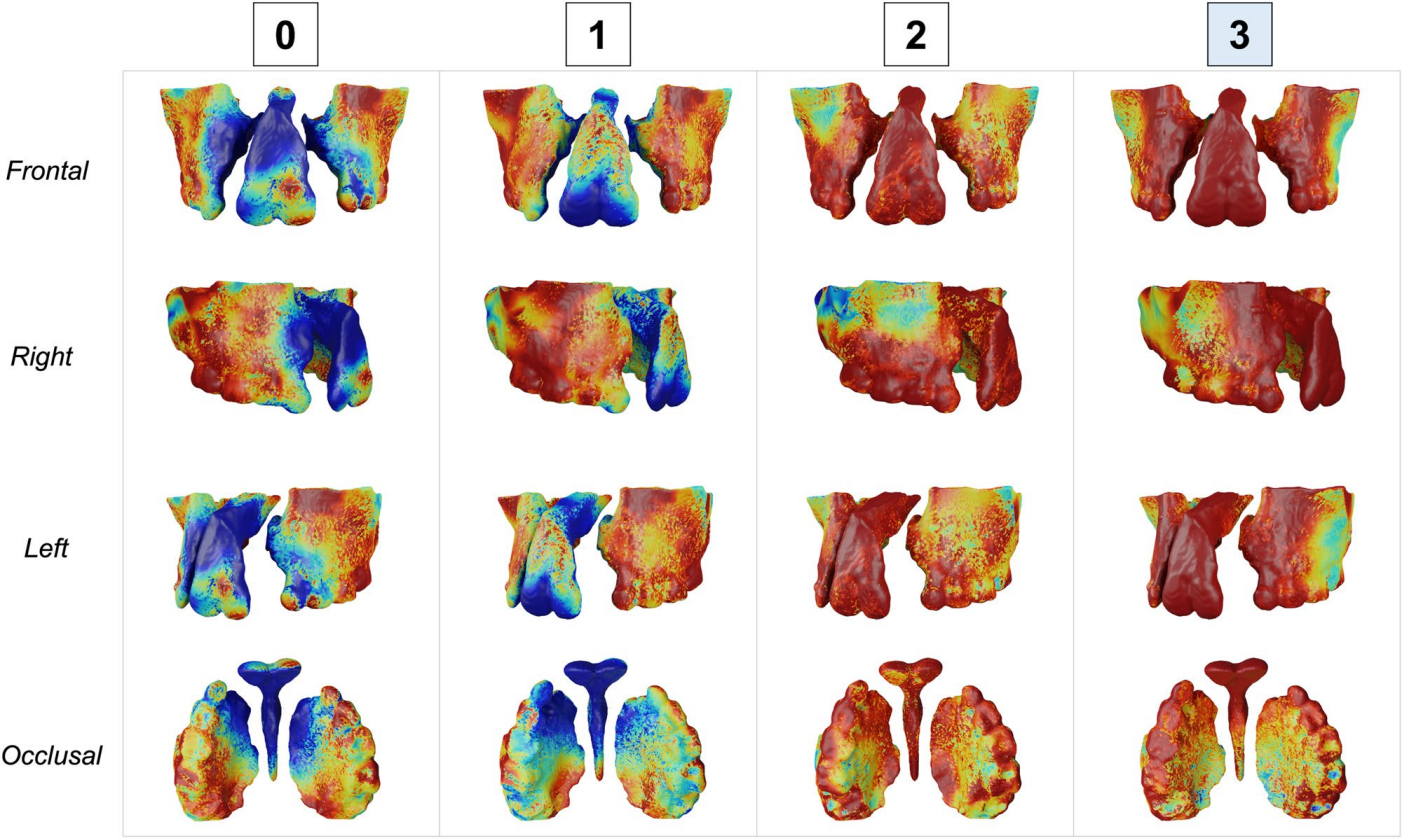
Explainability maps for a 3D surface model properly predicted as a class 3 of the severity index. In dark red are the most important features considered by the algorithm to output the classification, and in blue are the less important features. Note that in the heatmap for class 3, the premaxilla was considered the most important feature for this classification.

Figures 3, 4, 5 and 6 show the heatmaps generated by the SurfGradCam algorithm that graphically display the features used by the classifier. The heatmaps were generated by each different class (columns) in different view perspectives (rows). The color-coded graphic display ranges from blue to red, where in dark red are the most important features considered by the algorithm to output the classification, and in blue are the least important features. Figures 3, 4, 5 and 6 demonstrate that the algorithm was capable to adequately distinguish the most important features to determine each class in different models. The heatmap for class 0 focused correctly on the buccal and palatal aspect of the cleft side, with a dark red color in most of this region (Fig. 3). The dark red areas continuously decrease when assessing the same model for each class, with an increase in the blue areas (Fig. 3, second to third column). Note that Fig. 3 exemplify a model that was correctly predicted as class 0, and when the algorithm assessed this model as class 3 (Fig. 3, fourth column), most of the anterior region of the maxilla was displayed in dark blue, meaning that no important feature for this class was found in this 3D model. The same interpretation was found for different models predicted in each class. Figure 6 demonstrates a case that was correctly classified as class 3 and shows a dark red area emphasizing the projected pre-maxilla with the large alveolar bone defect and complete palatal discontinuity as the most important features for this classification. In the same figure, when the algorithm assessed for different classes, it becomes clear that no important feature was considered in the maxilla to classify this model as 0 or 1 (first and second column), and the different degree of red in the palate elucidates why this model was not classified as 2 (third column).

## Discussion

This is the first study to demonstrate a tool to automatically classify the alveolar bone defect of patients with CLP using an explainable algorithm. The use of AI technology has changed the 3D imaging analysis in the past years. The 3D assessment of the alveolar bone defect is an essential but complex task. For this reason, the use of AI-based models has increased with the purpose to simplify and increase the efficiency of this task. However, exist an increased concern regarding comprehending the full approach and reasons behind how an AI model predicts a decision^28–30^. Interpretable or explainable AI initiatives was proposed to promote more transparent AI models with more understandable outputs^28,29^. In this study, it was investigated through an interpretable AI algorithm which shape features were relevant to the classification/regression task for each training class in our data set by visualizing the key features directly on the 3D surface. Both the development of a classifier algorithm based on 3D surface evaluation of the alveolar bone defect of patients with CLP and the incorporation of an explainable algorithm are innovative and important advancements in the field.

The classifier task proposed in this study was able to accurately predict the severity of the alveolar bone defect in patients with CLP (Table 2). Multi-view shape analysis methods have been shown to be effective in a variety of tasks, including shape classification and retrieval, and they are particularly useful for 3D shapes that lack a clear orientation. The SurfGradCam algorithm was created to work directly on 3D surface models and was based on previously validated algorithms created for images or volumes^27,31–33^. The novelty of SurfGradCAM lies in its ability to provide a visual representation of the reasoning behind a neural network's classification decision for 3D shape models, which may support researchers to validate the shape features used in the task and address concerns related to the impact of machine learning systems on human lives. Figures 3, 4, 5 and 6 demonstrate that the algorithm is targeting the neighboring areas of the alveolar bone defect as the main features to predict the output. By visualizing the heatmap, researchers and clinicians can better understand the reasoning behind the classification decision and validate the shape features used in the task. This approach offers a novel way of explaining the decision-making process of a neural network and can be useful for addressing concerns related to the impact of machine learning systems on human lives.

In addition, a future clinical application includes the implementation of this classification task and interpretable algorithm in a clinical decision support system for planning the SABG in patients with cleft lip and palate. CDSS can combine clinical and imaging data to provide support for healthcare providers during the decision-making process. There is a growing interest in the implementation of CDSS in dentistry with different applications due to the usefulness and increase in the performance of AI algorithms^18,34–37^. Due to the complex and multidisciplinary face of the rehabilitation process of patients with CLP, the CDSS implementation would be beneficial by providing a standardized and full assessment of the alveolar bone morphology of patients with CLP before the SABG. This CDSS can be helpful in reducing treatment risks as well as providing relevant information necessary to surgical success. However, before this system can be effectively translated to a healthcare scenario, there is still a need for comparative effectiveness research that can provide the true value of AI and CDSS during the diagnosis and treatment planning^38^. A recent comparative effectiveness research application in Orthodontics showed that 3D image analysis and severity index promoted an overall change in responses of 43% regarding the diagnosis and treatment planning of impacted canines when compared to 2D image analysis^39^.

The 3D image analysis presents a more thorough assessment of bone morphology when compared to 2D assessments. However, it is still controversial in the literature the role of the morphology or shape of the cleft alveolar defect in the success of the surgical repair of the alveolar bone. A previous study showed a correlation between the morphology of the alveolar bone defect and SABG outcomes in patients with CLP using 3D imaging analysis^14^. In the future, other imaging inputs will also be included in this classifier index and a new model will be trained to be able to address different challenges and limitations that may influence the success of SABG in patients with CLP. The identification of treatment risks based on the alveolar bone defect characterization and tooth position near the alveolar cleft will be included as well as clinical data. A previous study has demonstrated the use of intraoral and extraoral 3D surface scans to satisfactorily design and print individualized appliances in patients with craniofacial disorders^40^. The future incorporation of AI technologies to automate this complex rehabilitation process will benefit these approaches even more. In addition, the use of optical scans for the creation of surface models that allows a 3D assessment of the facial characteristics are other important imaging input that can be added to assess and predict the severity of craniofacial morphology in patients with CLP.

Even though the use of AI algorithms has increased significantly, clinicians/experts are still required to supervise the outcomes of the AI predictions to allow new training and improve the performance of the models. As a limitation of this study, approximately 18% of the sample required a refinement of the alveolar bone defect segmentation after automatic segmentation. The refinements were necessary once that the alveolar bone defect shows a challenge and variable morphology for 3D automatic segmentation^15^. The alveolar bone defect morphology will vary from individual to individual, and therefore is a challenge to train an algorithm capable of segmenting these structures with a high level of accuracy. However, when compared to manual segmentation, automatic segmentation of the alveolar bone defect provides a less complex and time-consuming task^15^. New models with bigger samples are necessary to improve the performance of the segmentation algorithm. The clinician refinement allowed a new model to be trained to improve the performance of the AI segmentation tools for patients with CLP. This algorithm will be deployed as a new tool of the open-source 3D Slicer software^21^. In addition, future studies with larger samples should also be conducted to improve the robustness and performance of the segmentation algorithm for patients with CLP.

## Conclusion

A high overall precision, recall, F-1 score and AUC were observed for the classifier task. An overall accuracy of 0.816 was found for the automatic classification of the severity of the alveolar bone defect of patients with cleft lip and palate. The proposed interpretable AI algorithm showed a satisfactory level of accuracy to automatically predict the severity of the alveolar bone defect of patients with cleft lip and palate. In addition, the interpretable AI algorithm demonstrated adequately the correct features used for the severity index, focusing specifically on the regions neighboring the alveolar bone defect. Future applications will include the implementation of this new tool in a clinical decision support system to identify the treatment risks and favor surgical success. In addition, this automated severity index clinical decision support system can help in the diagnosis and treatment planning of presurgical orthodontics for patients with cleft lip and palate. The implementation of this clinical decision support system will incorporate other important imaging inputs and new models with larger sample will be trained to improve the robustness and performance of the algorithm.

## Data Availability

The data analyzed during the current study are available from the corresponding author on a reasonable request. The code and detailed read-me files used in this study are available in our GitHub repository (https://github.com/DCBIA-OrthoLab/Cl3ft). After additional validation, this algorithm will also be available for clinical and research use in an open-source web-based clinical decision support system (Smart-DOC: https://dsci.dent.umich.edu) and the 3D Slicer open-source platform (https://www.slicer.org/).
